# *Hippophae rhamnoides* reverses decreased CYP2D6 expression in rats with BCG-induced liver injury

**DOI:** 10.1038/s41598-023-44590-w

**Published:** 2023-10-13

**Authors:** Huiqiong Zou, Peipei Hao, Yingying Cao, Li Li, Ruifeng Ding, Xuefeng Bai, Yongzhi Xue

**Affiliations:** 1 Institute of Pharmacokinetics and Liver Molecular Pharmacology, Department of Pharmacology, Baotou Medical College, No. 31 Jianshe Road, Donghe District, Baotou, 014060 China; 2 Department of Gastroenterology, First Affiliated Hospital, Baotou Medical College, Baotou, China; 3 Department of Pathology, Baotou Cancer Hospital, Baotou, China

**Keywords:** Inflammation, Chronic inflammation, Immunology, Interleukins, Tumour-necrosis factors, Inflammatory diseases

## Abstract

In this study, we investigated the effect of *Hippophae rhamnoides* L. (HRP) on the activity of CYP2D6 via the CAMP/PKA/NF-κB pathway in rats with Bacille Calmette–Guerin (BCG)-induced immunological liver injury. BCG (125 mg/kg) was injected to establish the rat model of liver injury. HRP was administered intragastrically for one week as the intervention drug. Proteomics techniques were used to analyze protein expression levels, obtaining a comprehensive understanding of the liver injury process. ELISA or western blotting was used to detect specific protein levels. Dextromethorphan was detected using high-performance liquid chromatography to reflect the metabolic activity of CYP2D6. BCG downregulated the expression of CYP2D6, cAMP, PKA, IκB, and P-CREB and upregulated that of NF-κB, IL-1β, TNF-α, and CREB in the liver; HRP administration reversed these effects. Therefore, HRP may restore the metabolic function of the liver by reversing the downregulation of CYP2D6 through inhibition of NF-κB signal transduction and regulation of the cAMP/PKA/CREB/CYP2D6 pathway. These findings highlight the role of HRP as an alternative clinical drug for treating hepatitis B and other immune-related liver diseases.

## Introduction

*Hippophae rhamnoides* (HRP), a well-known medicinal and edible plant, also called sea buckthorn, is used as a new treatment strategy for injury and fibrosis of the liver, a major metabolic organ^1,2^. However, the sea buckthorn’s ability to regulate imbalanced metabolic enzymes associated with liver diseases that cause immune-mediated liver damage, such as viral hepatitis, is not fully understood. Viral hepatitis accounts for more than half of the incidence of liver diseases^3^. Among the immune factors related to liver injury, hepatitis B infection is the main cause of chronic liver disease globally^4,5^, affecting 257 million people worldwide^6^. Inadequate treatment of chronic liver injury can lead to liver fibrosis, liver dysfunction, and cancer^7^. The immune system’s fight against the virus impairs liver cells and liver function^8^. Immune liver injury is caused by specific antiviral T cell-mediated immunity^9^, which induces inflammatory cell infiltration and elevated expression of inflammatory cytokines, leading to liver tissue damage. It also decreases drug metabolism and disposal ability, resulting in increased exposure to metabolized substrate drugs, adverse reactions, and drug interactions^10–12^. Cyclic adenosine-3,5′-monophosphate (cAMP) is an important mediator of the inflammatory response^13^. cAMP triggers protein kinase A (PKA), which phosphorylates cAMP reactive element binding protein (CREB)^14^. CREB is then translocated to the nucleus where it promotes the synthesis of pro-inflammatory mediators and anti-inflammatory cytokines, stimulating macrophage polarization, exocytosis, and granulocyte apoptosis, ultimately alleviating inflammation^15^. PKA also decreases the transcriptional activity of NF-κB, thereby preventing the expression of inflammatory genes. The activation of the cAMP signaling pathway is a promising pharmacological strategy for treating inflammatory diseases by promoting inflammatory regression^13,16^. As a natural therapy, traditional Chinese medicine has the dual benefit of treating diseases while maintaining health, with unique advantages such as safety, low toxicity, and high efficiency^17^. Extracting the active components of natural medicinal plants for the treatment of diseases is highly popular, among which, the sea buckthorn is a promising candidate^18^.

The sea buckthorn of the *Elaegenidae* genus is known worldwide for its nutritional, anti-inflammatory, antioxidant, and liver-protecting properties^19,20^. Sea buckthorn berries are rich in nutrients and compounds, such as vitamins, carotene, flavonoids, essential oils, carbohydrates, organic acids, amino acids, and minerals^21,22^. Flavonoids and oil extracts of the sea buckthorn have immunoregulatory, antioxidant, and hepatoprotective abilities^19^. Furthermore, sea buckthorn preparations have been widely used in the treatment of gastric diseases, cardiovascular diseases, liver injury, and skin diseases^19^. Experiments have shown that pre-treatment with sea buckthorn protected mice from liver injury induced by lipopolysaccharide/D-galactosamine^19^. Clinical studies have confirmed that the use of sea buckthorn reduces the levels of serum laminin, hyaluronic acid, total bile acid, and types III and IV collagen in patients with cirrhosis, indicating its role in restricting the synthesis of collagen and other extracellular matrix components^23^. Therefore, the sea buckthorn may be an effective drug for the prevention and treatment of acute liver injury and fibrosis.

**Figure 1:**
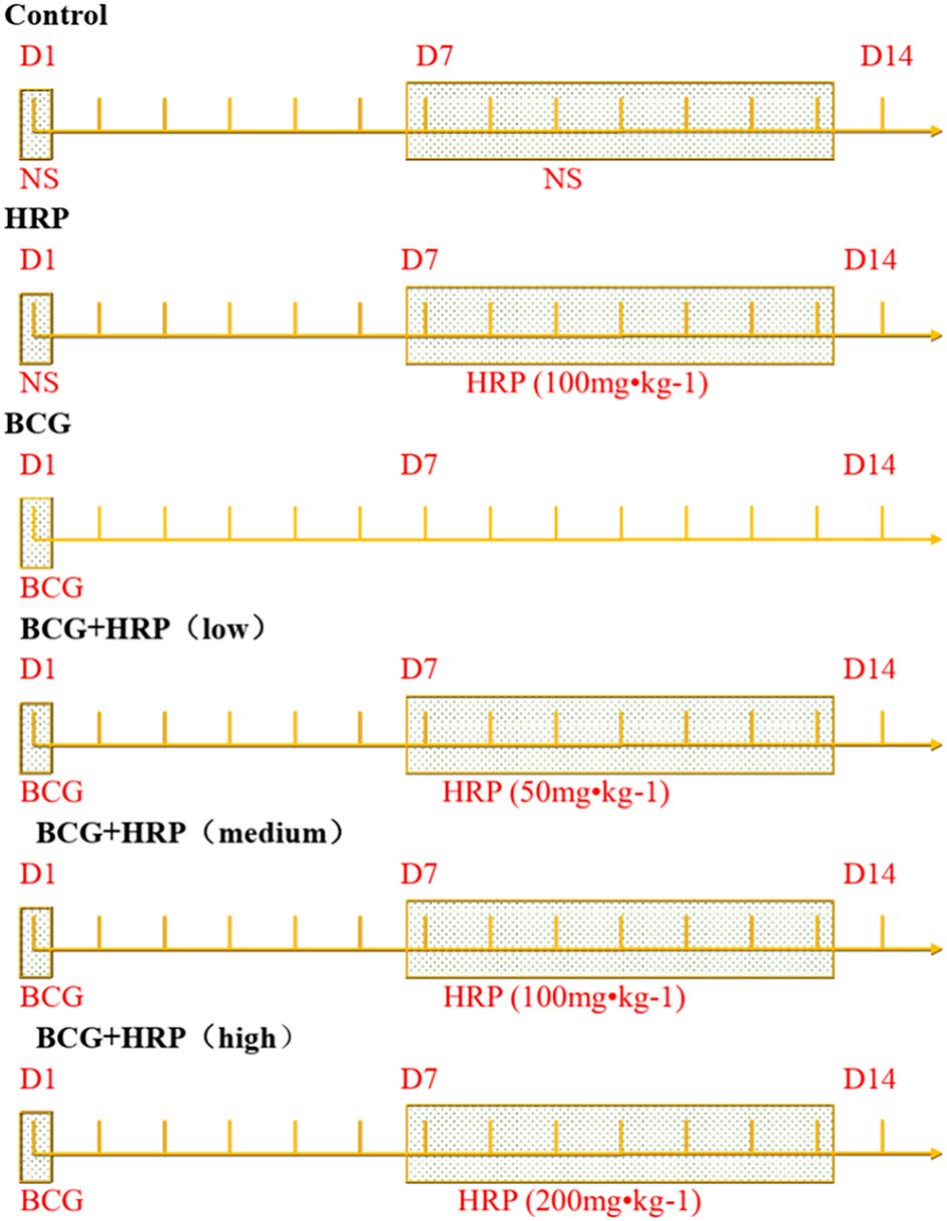
Experimental design and grouping.

CYP2D6 is mainly expressed in the liver and accounts for 1.3–4.3% of the total amount of CYP450 (CYP). It can metabolize at least 160 drugs^24^, which accounts for 20–30% of all clinically used drugs^25–27^. Despite its relatively low expression level, CYP2D6 is considered an important CYP owing to its involvement in numerous metabolic pathways of drugs and exogenous substances. At present, most studies on the metabolic function of CYP2D6 have focused on gene polymorphism, with its changes and regulatory mechanisms involved in the process of immune liver injury remaining unclear. We previously demonstrated the regulatory effect of NF-κB on CYP2E1 in Bacille Calmette–Guerin (BCG)-induced immune liver injury^28^. The sea buckthorn was also shown to reverse the downregulation of CYP3A by inhibiting NF-κB signal transduction in cases of immune liver injury^1^. The mRNA expression of CYP2D6 strongly correlated with enzyme activity in liver tissues of more than 130 non-weak metabolizers (r = 0.95), similar to that of CYP3A4 (r = 0.90)^25^. These findings suggest that sea buckthorn may reverse the downregulation of CYP2D6 expression by inhibiting NF-κB signal transduction during immune-mediated liver injury. During the process of immune liver injury, the changes in CYP2D6 and its regulation by HRP may be similar to those of the CYP subtypes 2E1 and 3A, which have been previously studied, or there may be new regulatory mechanisms. In this study, we evaluated the changes in metabolic activity and protein expression of CYP2D6 and the effect of HRP on the cAMP/PKA/NF-κB pathway in an immune-mediated liver injury rat model obtained by injecting BCG into the tail vein of rats. Moreover, we further explored the effects and mechanisms of action of HRP on CYP2D6 in immune-mediated liver injury to identify new targets and uncover new hypotheses for drug therapy (Fig. 1).

## Results

### Effect of HRP on BCG-induced liver injury

**Figure 2:**
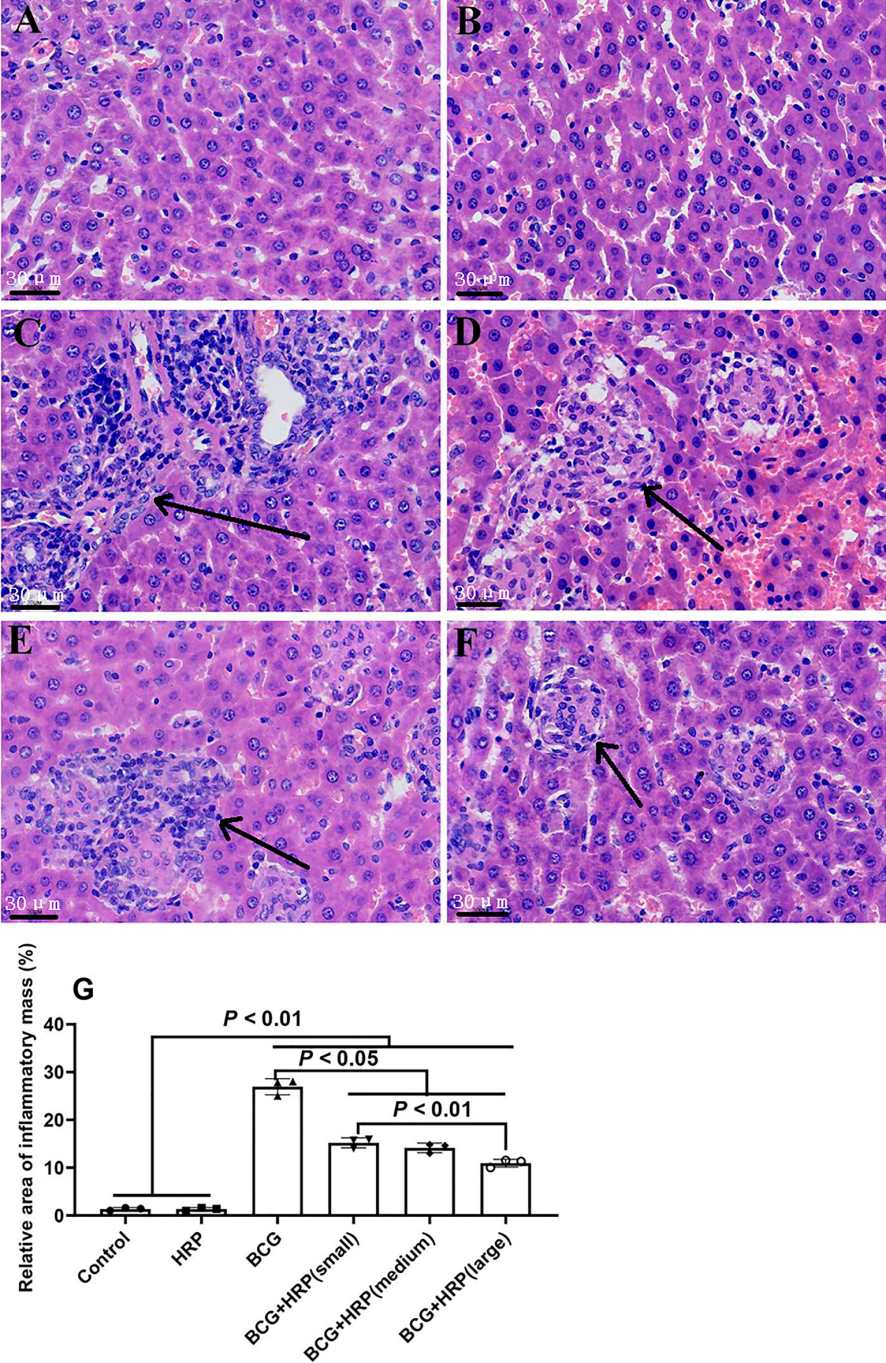
Effects of HRP on liver pathology of rats with BCG-induced immune-mediated liver injury. (**A**–**F**) Representative light microscope images of rat livers of the (**A**) control group (H&E staining; original magnification, × 200); (**B**) HRP group; (**C**) BCG group (arrows point to a granuloma made up of inflammatory cells); (**D**) BCG + HRPL group (50 mg⋅kg^−1^); (**E**) BCG + HRPM group (100 mg⋅kg^−1^); and (**F**) BCG + HRPH group (200 mg⋅kg^−1^). (**G**) Representative light microscope images of areas with inflammatory cell infiltration (S) in the liver in each group. Data are expressed as the mean ± standard deviation (SD) (n = 6 rats). *BCG* Bacille Calmette-Guerin; *HRP Hippophae rhamnoides; HRPL* HRP low-dose group; *HRPM* HRP medium-dose group; *HRPH* HRP high-dose group; *H&E* Haematoxylin and eosin.

**Figure 3:**
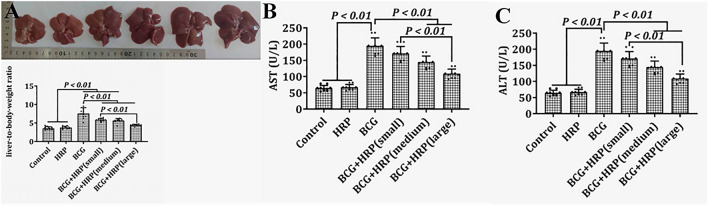
Effects of HRP on the liver weight or biochemical changes of rats with BCG-induced immune-mediated liver injury. (**A**) Rat liver weight; (**B**) Rat serum ALT; (**C**) Rat serum AST. Data are expressed as the mean ± standard deviation (SD) (n = 6 rats). *BCG* Bacille Calmette-Guerin; *HRP Hippophae rhamnoides*; *ALT* Alanine aminotransferase; *AST* Aspartate aminotransferase.

H&E staining showed a normal histological structure of the liver tissues of control rats (Fig. 2A). The morphological characteristics of the liver in the HRP group were normal and similar to those in the control group (Fig. 2B). In contrast, in the BCG group, the hepatic parenchyma and portal vein area were surrounded by a large number of monocytes and lymphocytes, forming diffuse inflammatory masses of different sizes. The structure of the hepatic cord was unclear, and the liver cells showed vacuolar degeneration (Fig. 2C). After BCG stimulation, the addition of HRP considerably improved liver histology in a dose-dependent manner (Fig. 2D–F). Three visual fields were randomly selected from each liver section in each group. The relative area (S) of the infiltrated area of inflammatory cells is shown in Fig. 2G. Compared with that in the control group, S in the BCG group was significantly increased (*P* < 0.05). Similarly, HRP intervention reduced BCG-induced immune-mediated liver injury in rats in a dose-dependent manner (*P* < 0.05). Compared with that in the control group, the liver weight in the BCG group was significantly increased (*P* < 0.05). However, HRP inhibited the increase in liver weight after BCG stimulation in a dose-dependent manner (*P* < 0.05; Fig. 3A). AST and ALT levels were significantly (*P* < 0.05) elevated in the BCG group relative to the control. However, the HRP treatment in BCG-stimulated rats caused a significant decrease (*P* < 0.05) in AST and ALT levels compared to the same treatment in the control rats (Fig. 3B and C, respectively).

### Effect of HRP on rat liver proteomics

**Figure 4:**
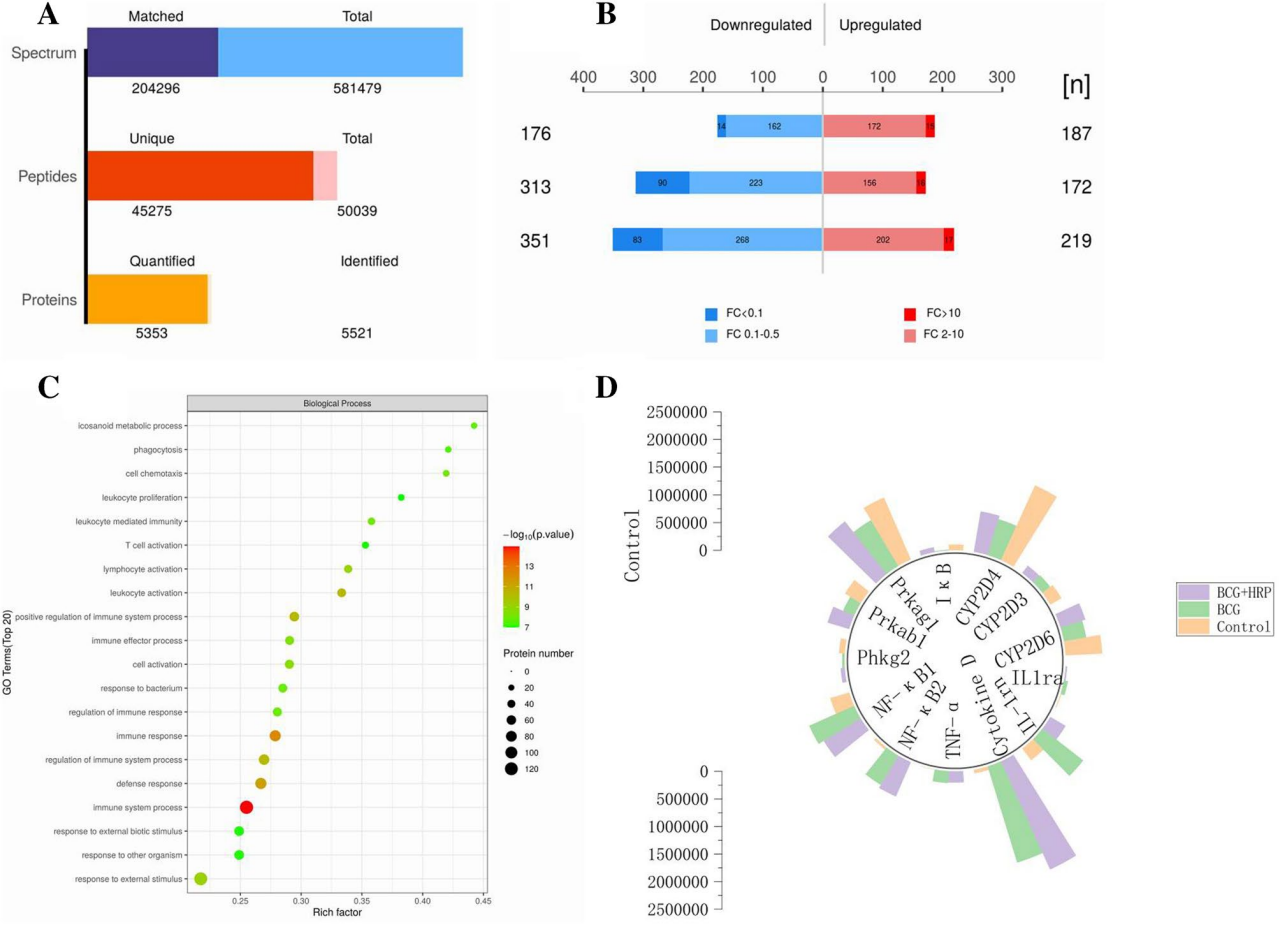
Effects of HRP on rat liver proteomics. (**A**) Statistical map of omics identification results. (**B**) Histogram of the total number of different proteins in the control group and the BCG group (**C**) GO (Gene Ontology, GO) functional enrichment bubble map (BP) in the biological process in the control versus BCG groups. GO functional enrichment bubble diagram; the closer the bubble to the color red, the stronger the significance, and the larger the bubble, the higher the number of proteins. (**D**) Histogram of protein quantitative difference results. *BCG* Bacille Calmette-Guerin; *HRP Hippophae rhamnoides*. cytochrome P450, family 2 (Cyp2d6, Cyp2d3, Cyp2d4), inhibitor of nuclear factor kap (Ikbkb); protein kinase AMP-activated (Prkag1); protein kinase AMP-activated (Prkab1); phosphorylase kinase catalytic (Phkg2); nuclear factor kappa B subunit (Nfkb1); nuclear factor kappa B subunit (Nfkb2); TNF alpha induced protein (TNF-α); dedicator of cytokine (cytokine D); interleukin 1 receptor antagonist (Il-1rn); interleukin 1 receptor access (Il-1ra).

Proteomics techniques were used to analyze the liver proteins of rats in the control, BCG, and HRP intervention groups, and the results are shown in Fig. 4. A total of 5521 proteins were detected in the liver of rats in the control, BCG, and BCG + HRP (medium) groups, and 5353 proteins were quantified (Fig. 4A). The number of highly and poorly expressed proteins varied between the groups, with the darker regions indicating the number of significantly differentially expressed proteins (*P* < 0.05, Fig. 4B). The GO functional enrichment bubble diagram revealed that the BCG group had an enhanced immune response (Fig. 4C). Among the differentially expressed proteins, the subtypes of metabolic enzymes and their regulatory pathway proteins are discussed in this study. Furthermore, the protein quantification analysis demonstrated that CYP2D, PKA, and phosphorylated kinase were downregulated in the BCG group compared with those in the control group, and HRP administration could partially restore their expression levels. HRP intervention also inhibited the activation of the NF-κB, IL-1β, and TNF-α pathways in the BCG group (Fig. 4D).

### Effect of HRP on CYP2D6 metabolic activity

**Figure 5:**
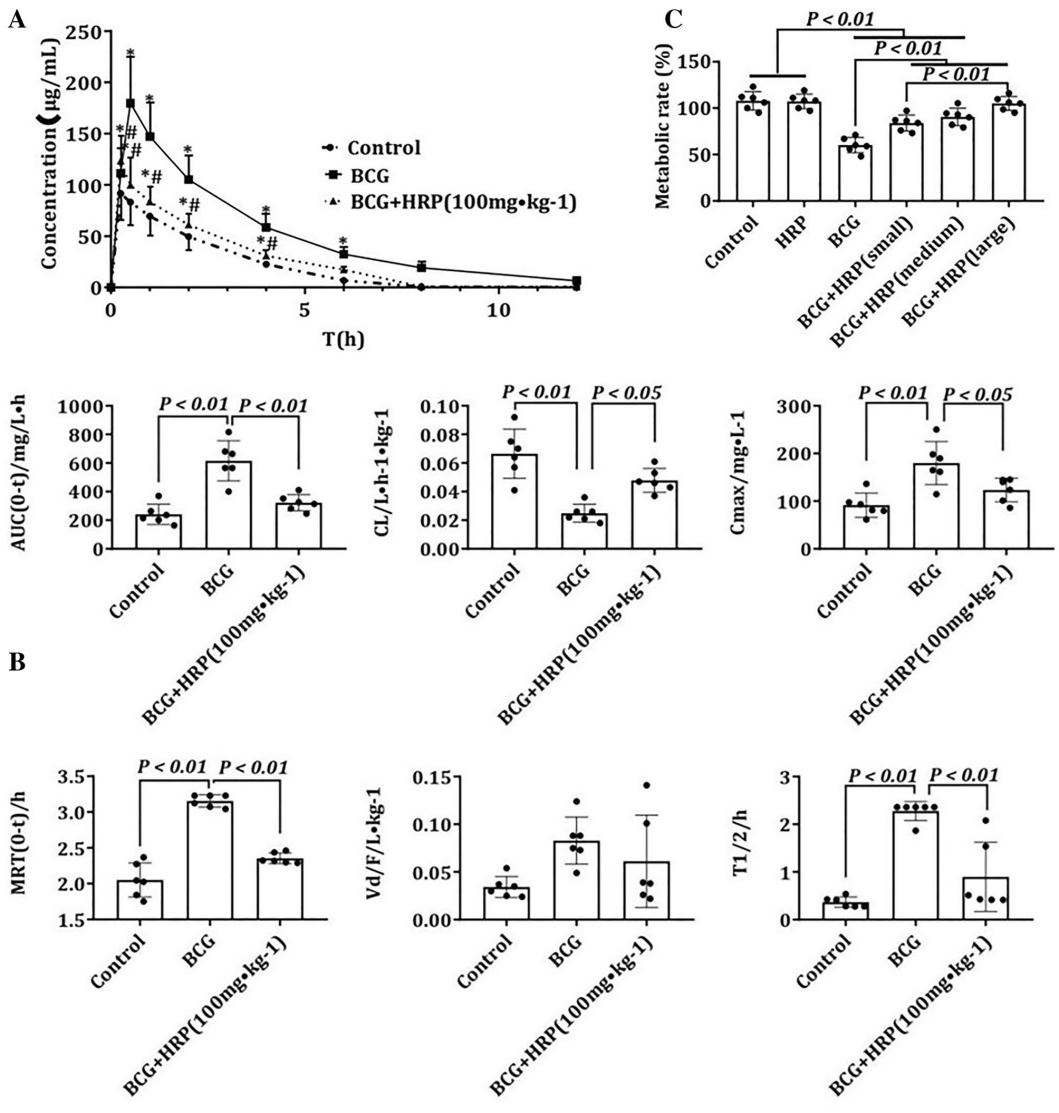
Effects of HRP on the metabolic activity of CYP2D6 in rats with BCG-induced immune-mediated liver injury. (**A**) Blood samples were collected 0.25, 0.5, 1, 2, 4, 6, 8, and 12 h after DEM administration. Plasma dextromethorphan was measured using HPLC as described. Data are expressed as the mean ± standard deviation (SD), n = 6. *P < 0.01 compared with the control group. # *P* < 0.01, compared with the BCG group. Significance was determined by one-way analysis of variance (ANOVA), followed by Tukey’s test. (**B**) Parameters (AUC, CL, C_max_, MRT, Vd, and T1/2) were calculated from the plasma dextromethorphan concentration curve as described. Data are expressed as the mean ± SD (n = 6 rats). (**C**) Metabolic rate was expressed by the amount of dextromethorphan metabolized after incubation with microsomes in vitro and was normalized according to the control group. Data are expressed as the mean ± SD (n = 6 rats). *AUC* Area under the curve; *BCG* Bacille Calmette-Guerin; *CL* Clearance rate; *C*_*max*_ Maximum blood concentration; *DEX* Dextromethorphan; *HPLC* High-performance liquid chromatography; *HRP Hippophae rhamnoides*; *MRT* Resident time in vivo; *T1/2* Half-life; *Vd* Apparent volume of distribution.

The plasma DEX concentration in rats was measured using high-performance liquid chromatography (HPLC). These concentrations at 0.5, 1, 2, 4, and 6 h in the BCG group were higher than those in the control group and lower in the BCG + HRP group than those in the BCG group (*P* < 0.05) (Fig. 5A). According to the pharmacokinetic parameters calculated using DAS3.0 software, AUC, C_max_, MRT, and T1/2 were significantly higher and CL was significantly lower in the BCG group than in the control group (*P* < 0.05). HRP intervention reversed the changes in metabolic parameters. Vd did not differ among the three groups (Fig. 5B).

In vitro microsomal incubation experiments showed that the metabolic activity of rat microsomes was similar to that of the control group treated with HRP alone. Immune stimulation with BCG reduced the metabolic activity of DEX metabolized by CYP2D6, and HRP intervention restored the metabolic activity of CYP2D6 in a dose-dependent manner (*P* < 0.05) (Fig. 5C).

### Effect of HRP on protein expression

**Figure 6:**
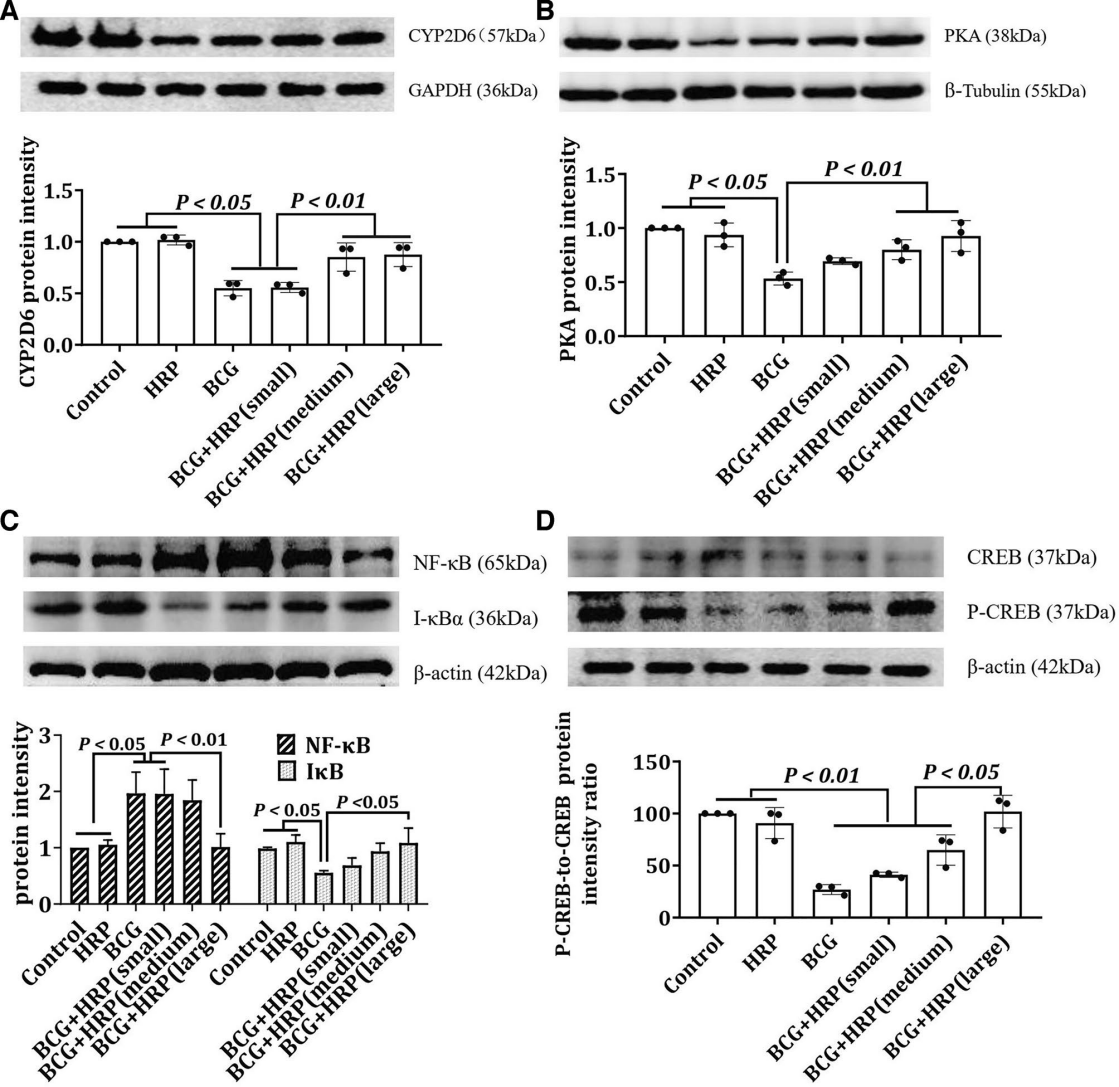
Effect of HRP on the expression of CYP2D6, PKA, CREB, PCREB, IκB, and NF-κB in rats with BCG-induced immune-mediated liver injury. Rats were administered BCG (single intravenous dose of 125 mg/kg BCG) or BCG + HRP (50, 100, or 200 mg⋅kg^−1^⋅d^−1^ orally for 7 d). Liver proteins were extracted to determine the expression levels of CYP2D6, PKA, CREB, PCREB, IκB, and NF-κB. SDS-PAGE was performed using equal amounts (30 μg) of protein, and western blotting was performed using antibodies against CYP2D6, PKA, CREB, PCREB, i-κB, and NF-κB. The results were normalized to tubulin, GAPDH, or β-actin. The protein expression levels of CYP2D6 (**a**), PKA (**b**), IκB and NF-κB (**c**), CREB, and P-CREB (**d**) in the rat liver were measured by western blotting. The expression levels of CYP2D6, PKA, IκB, NF-κB, CREB, and P-CREB were quantified using the ImageJ software (NIH, Maryland, USA). The data represent the mean ± standard deviation (SD) of three independent experiments. *BCG* Bacille Calmette-Guerin; *HRP Hippophae rhamnoides.*

Western blot analysis revealed uniform density between bands compared to the endogenous tubulin or β-actin proteins. Thus, protein quantification was proven accurate, and the amount of protein in the sample was consistent (Fig. 6). Compared with that in the control or HRP groups, NF-κB and CREB in the BCG group were appreciably upregulated. However, HRP intervention inhibited the overexpression of NF-κB and CREB in a dose-dependent manner, and the expression of IκB showed the opposite trend to that of NF-κB (Fig. 6; *P* < 0.05). The levels of CYP2D6, PKA, and the phosphorylated product of CREB (P-CREB) were noticeably reduced in the BCG group. However, HRP intervention partially restored the expression of CYP2D6, PKA, and P-CREB to control levels in a dose-dependent manner (Fig. 6c; *P* < 0.05).

### Contents of cAMP, TNF-α, and IL-1β

**Figure 7:**
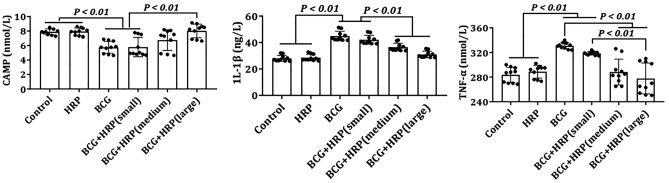
Effect of HRP on cAMP, TNF-α, and IL-1β levels in rats with immune-mediated liver injury. Rats were administered BCG (single intravenous 125 mg/kg dose of BCG) or BCG + HRP (50, 100, or 200 mg⋅kg^−1^⋅d^−1^ orally for one week). Each bar represents mean ± standard deviation (SD) of three independent experiments (n = 10 per group). *BCG* Bacille Calmette-Guerin; *HRP Hippophae rhamnoides.*

As shown in Fig. 7, pro-inflammatory cytokines (TNF-α and IL-1β) were significantly upregulated 14 d after BCG injection compared to the control group. However, HRP preconditioning inhibited the upregulation of TNF-α and IL-1β in a dose-dependent manner (*P* < 0.05). The levels of cAMP were considerably reduced in the BCG group. However, HRP intervention partially restored the expression of cAMP to control levels in a dose-dependent manner (*P* < 0.05). Compared to those in the control group, the cAMP, TNF-α, and IL-1β levels in the HRP group showed no significant changes.

## Discussion

The liver proteomics analysis conducted in this study reflected the changes in liver protein composition in the control, BCG, and HRP intervention groups after BCG injury and immune response activation and corresponding regulatory proteins. Further analysis showed that BCG immune stimulation led to the downregulation of CYP2D, PKA, and phosphoryl-kinase, which could be partially restored after HRP administration. Therefore, HRP intervention inhibited the activation of NF-κB, IL-1β, and TNF-α pathways; pharmacokinetic and molecular pharmacological experiments further confirmed these results.

Immuno-stimulation with BCG downregulated liver CYP2D6, and HRP intervention reversed this effect in a dose-dependent manner to protect liver metabolic function. Sea buckthorn granules are mainly composed of sea buckthorn fruits rich in vitamins, carotenoids, flavonoids, essential oils, carbohydrates, organic acids, amino acids, and minerals^1^. HRP administration alone did not exhibit any appreciable effect on the expression and metabolic activity of liver CYP2D6, indicating that HRP does not normally induce or inhibit CYP2D6. CYP2D6 is an important drug-metabolizing enzyme primarily expressed in the liver, where it oxidizes and metabolizes over 20% of clinically used drugs^12^. Its expression and activity directly affect the exposure of metabolized drugs in the body, resulting in changes in drug efficacy, adverse drug reactions, and increased drug interactions, thereby affecting the therapeutic effect^29^. The US Food and Drug Administration recommends the use of DEX as a sensitive probe for detecting CYP2D6 activity in rats and humans, both in vitro and in vivo^30^. CYP2D6 metabolizes approximately 96% of DEX to dexphenol alkane, while the remaining 4% is metabolized to other metabolites by CYP3A4^30,31^. Therefore, the amount of DEX metabolized can be utilized to evaluate the metabolic activity of liver CYP2D6. Here, we describe an additional mechanism of the hepatoprotective effect of HRP, which is regulating imbalanced CYP2D6 associated with immune-mediated liver injury, restoring liver metabolic capacity, and eliminating harmful substances via metabolism. To investigate the mechanism of action of HRP administration on CYP2D6 in the liver of rats with BCG-induced immune-mediated liver injury, we further explored the cAMP/PKA/NF-κB pathway. We established a BCG-induced rat liver inflammation model and administered various doses of HRP, resulting in inflammation regression. The gradient changes in IL-1β, TNF-α, NF-κB, cAMP, PKA, CREB, and CYP2D6 during the inflammatory process, along with the occurrence and remission of inflammation, suggest that IL-1β may be involved in the pathological process of hepatitis. The IL-1β/TNF-α/NF-κB pathway is activated during the inflammation initiation process, and HRP can downregulate its activation in a dose-dependent manner, implying that this pathway is involved in the initiation of hepatitis. Furthermore, immune stimulation with BCG led to the downregulation of the cAMP/PKA/P-CREB/CYP2D6 pathway, and the activity of the pathway was restored in a dose-dependent manner after exposure to HRP, suggesting that this pathway is involved in the remission of inflammation.

cAMP is a part of the endogenous mechanism that downregulates the inflammatory response and prevents the progression of acute inflammation, allowing for the beneficial effects of chronic inflammation and its associated tissue destruction^24^. When liver cells are stimulated by BCG-induced immune damage, receptor TRL4, located on the cell membrane, recognizes the harmful stimulus, activates the signaling cascade, and transmits information to the nucleus through the activation of NF-κB^32^; this triggers the production of pro-inflammatory mediators, proteases, and reactive oxygen species and increases the expression of adhesion molecules on the cell surface^11^. cAMP-mediated activation of PKA is triggered by the binding of cAMP to the regulatory subunit of PKA, which changes the protein conformation, releases the catalytic subunit to phosphorylate, and activates the transcription factor CREB^11^. P-CREB promotes the expression of anti-inflammatory cytokines (such as IL-10)^33^ and decreases TNF-α levels^34^. The effect of cAMP on the regulation of proinflammatory mediators is mediated by PKA, leading to NF-κB-induced transcriptional inhibition^35^. cAMP/PKA activation prevents the degradation of IκB, thereby preventing NF-κB translocation^36^ and inhibiting the signaling pathway leading to the activation of NF-κB, which, in turn, inhibits the pro-inflammatory mediators TNF-α and IL-1, thus promoting the release of anti-inflammatory molecules^37^. The treatment of MTB-infected macrophages with lysophosphatidylcholine (LPC) has been shown to increase cAMP levels and activate PKA to promote phagosome maturation^38^, which also reduces NF-κB levels and leads to decreased secretion of pro-inflammatory cytokines and increased production of anti-inflammatory cytokines. Our experimental findings showed that in the phase of inflammation regression, HRP inhibited NF-κB by upregulating PKA activated by cAMP and inhibited TNF-α and IL-1β, which resulted in liver inflammation regression and restoration of damaged CYP2D6 and liver function. In this study, BCG immunostimulation induced hepatitis, characterized by inflammatory cell infiltration to form large and small clumps, increased liver weight, increased TNF-α and IL-1β, and increased AST/ALT. HRP administration could reverse these changes in a dose-dependent manner. In terms of pathological manifestation, inflammation in the BCG group was more typical, while that in the HRP intervention group gradually subsided. cAMP/PKA/P-CREB decreased in the BCG group and recovered in a dose-dependent form in the HRP group, with consistent trends observed in western blotting and proteomics results. cAMP/PKA/P-CREB decreased during hepatitis triggering and increased during hepatitis recovery, providing further evidence that cAMP is an important secondary messenger during hepatitis regression. However, no significant changes in TRL-4 levels were observed during this process, possibly because our model reflects the recovery state of inflammation rather than its initiation stage; therefore, this finding indicates that TRL-4 primarily acts during the initiation stage. Furthermore, the observed trend shows that both CYP2D6 and CYP3A undergo similar changes and regulation mechanisms during the immune liver injury process, and HRP has a comparable intervention effect in this pathology. Indeed, these two subtypes of metabolic enzymes are closely related to clinical drugs in the CYP family and metabolize different types of clinical drugs.

Notably, the pathways discussed in this study exhibit different manifestations at different stages of hepatitis and inflammation. The correlation is not linear, but rather a complex and interactive network connection between multiple factors. In addition, a specific limitation of this study is that rats cannot be naturally transfected with human hepatitis virus^39–41^. Although we successfully replicated a rat model of immune-mediated liver injury induced by the BCG vaccine, which has been recognized by scholars both domestically and internationally as a model simulating immune liver injury caused by viral hepatitis, there are certain species differences and model limitations. Therefore, transgenic rats should be used in subsequent studies to reduce species differences, and targeted proteomics should be employed to observe the overall disease process and further explore molecular mechanisms. Additionally, as sea buckthorn granules are widely used in Mongolian and Han medicine and are traditionally consumed as both drugs and food, adverse reactions associated with them have been relatively few and can be further confirmed by clinical trials.

In conclusion, we determined that HRP mainly inhibits the downregulation of CYP2D6 during immune-mediated liver injury through transcriptional regulation of cAMP/PKA/NF-κB. In addition to NF-κB, cAMP is a notable molecular target. The expression and activity of CYP2D6 directly affect the efficacy, adverse drug reactions, and drug interactions of over 160 clinical drugs, including antipsychotics, antidepressants, antiemetic drugs, antihistamines, analgesics, cancer drugs, and antiarrhythmic agents^24^. According to this study, in the process of immune-mediated liver injury, the drug regimens in these fields can be adjusted reasonably to achieve precise treatment and individualized medication. This mechanism also applies to alcoholic liver injury and non-alcoholic fatty liver disease. These findings will help enrich the current understanding of the clinical effects of sea buckthorn granules and provide new strategies for hepatitis treatment.

## Methods

### Animals

Six-week-old male SD rats (weight, 200 ± 20 g; License No. SCK (Beijing) 2019-0010) were obtained from SPF Biotechnology Co., Ltd. (Beijing, China). After one week of adaptive feeding, the rats were randomly divided into six groups (n = 12): blank control, HRP, BCG, and three BCG + HRP groups (low-, medium-, and high-dose). The rats were housed at 25 ± 1 °C with a 12-h light/dark cycle and access to water and food ad libitum. All procedures were performed during the light phase. All animals were handled humanely, and experiments were conducted in accordance with the ARRIVE and other relevant guidelines and regulations. The Ethics Committee of Baotou Medical College approved the research program (Approval number: 2021-061). The rats in the model group were administered a single injection of the *M. bovis* BCG vaccine (China Ruichu Biotechnology Co., Ltd., Shanghai, China, 125 mg/kg) through the tail vein, while the rats in the control and HRP groups were injected with the same amount of normal saline (NS). After one week, rats in the control and BCG groups were intragastrically administered NS (100 mg/kg) every day for one week. Rats in the BCG + HRP groups were administered sea buckthorn granules (Sichuan Meidakang Pharmaceutical Co., Ltd., Sichuan, China, Batch number: 20200415) daily at the following doses: low (50 mg/kg), medium (100 mg/kg), and high (200 mg/kg). Rats in the HRP group were administered BCG + HRP (medium dose, 100 mg/kg) for one week. BCG induction lasted for two weeks, HRP intervention lasted for one week with fasting the night before, and all procedures were performed at 8:00 a.m. on day 14. Half of the rats in each group were used to analyze the metabolic activity of CYP2D6 in vivo, while the other half were sacrificed for liver decapitation, weighed, and used for liver hematoxylin and eosin (H&E) staining and follow-up experiments. After the experiment, all rats were anesthetized with excess pentobarbital sodium and decapitated. The experimental design is illustrated in Fig. 1.

### Histological analysis of the liver

The liver tissues from the rats were fixed in 10% neutral buffered formalin, embedded in paraffin, cut into 5-μm thick tissue sections, stained with H&E, and examined using an Olympus CX23 microscope (Tokyo, Japan). A Hamamatsu Pathological Biopsy Scanner (C13140-01, Hamamatsu Photonics Co., LTD., Tokyo, Japan) was used to prepare the digital slides. Under the guidance of pathologists, NDP.view2 U12388-01 digital pathological section scanning software (Hamamatsu Photonics Trading (China) Co., LTD., Beijing, China) was used to draw the boundary of inflammatory cell clusters and display the area of the clusters. Three fields of view were selected for measurement for each sample, and the average value was taken to determine the relative area of inflammatory cell clusters.

### Serum AST and ALT determination

Sera were collected 14 d after BCG injection, and serum ALT and AST levels were measured using an automated biochemical analyzer (Hitachi 7600-020, Tokyo, Japan).

### LC-MS/MS proteomics profiling

Proteomics profiling was conducted at Shanghai Applied Protein Technology Co., Ltd, using liquid chromatography-tandem mass spectrometry (LC-MS/MS)^29^. Additional information regarding protein extraction and digestion, optional fractionation, chromatographic conditions and MS parameters, protein identification, quantitation, and bioinformatic analysis are available in the Supplementary Materials.

### In vivo* metabolism kinetics of DEX*

On day 14 of the experiment, six rats in the control, BCG, and BCG + HRP groups were administered dextromethorphan (DEX) hydrobromide syrup (15 mg·kg^−1^; Jiangxi Pharmaceutical Durenhe Pharmaceutical Co., Ltd., Jiangxi, China) by gavage. Blood was collected from the inner canthus vein using a capillary glass tube (diameter, 0.8 mm) at various time intervals after administration (0.25, 0.5, 1, 2, 4, 6, 8, and 12 h). The collected blood was centrifuged at 3000 g for 10 min to obtain plasma. DEX levels were determined using high-performance liquid chromatography (HPLC; Waters 2998 Photodiode Array Detector, Massachusetts, USA)^30^. Plasma concentration-time curves were plotted using the pharmacokinetic software DAS3.0 (Chinese Committee of Mathematical Pharmacology, Shanghai, China). The pharmacokinetic parameters area under the curve (AUC), clearance rate (CL), maximum blood concentration (C_max_), resident time in vivo (MRT), apparent volume of distribution (Vd), and half-life (T1/2) were obtained in the non-atrioventricular model as indicators of CYP2D6 activity in vivo.

### In vitro* metabolism of DEX*

The metabolism of DEX in liver microsomes of rats pre-treated with drugs in vitro was used as an indicator of CYP2D6 activity. Liver microsomes were prepared using a calcium precipitation method. Briefly, the rat liver was thawed on ice, homogenized in phosphate buffer, and centrifuged at 12,000 g for 20 min; the supernatant was added to 88 mM CaCl_2_ (supernatant: CaCl_2_ = 1:1), mixed and combined, and centrifuged at 15,000 g for 30 min. After two rounds of centrifugation, the resulting precipitates were added to phosphate buffer for re-suspension to obtain microparticles. Liver microsomes (100 μL), an appropriate amount of DEX, 1 mM of NADPH, and 0.1 M of phosphate buffer (pH 7.4) were added to the reaction system for a total volume of 200 μL. The reaction mixture was pre-incubated at 37 °C for 5 min, followed by the addition of NADPH to initiate the reaction and incubation for 30 min. The reaction was terminated with 600 μL of glacial ethyl acetate. DEX metabolism in the reaction system was analyzed using HPLC.

### ELISA

Rat liver tissue (0.2 g) was weighed, and 0.1 M PBS (pH 7.4) was added for homogenization. The mixture was centrifuged at 10,000 g for 10 min, and the supernatant was collected for analysis. The levels of TNF-α, IL-1β, and cAMP in the liver tissues were determined using an ELISA kit (Jiangsu Enzyme Free Industry Co., Ltd., Jiangsu, China) according to the manufacturer's instructions.

### Western blotting

The liver tissues of each experimental rat were weighed and mixed with NP-40 lysate, broad-spectrum protease inhibitor, and 0.1 M EDTA homogenate. The mixture was lysed on ice for 30 min and centrifuged at 10,000 g for 10 min; the supernatant was collected to obtain the total tissue protein. An extractor kit (Boster Biological Technology, Wunan, China; Catalog number: AR0106) was used to extract nuclear and cytoplasmic proteins, according to the manufacturer's instructions. After separating the sample protein by 10% SDS-PAGE, it was transferred onto a PVDF membrane (Boster Biological Technology, Wunan, China; Catalog number: AR0136-04). Primary antibodies against p450 2D6 (dilution 1:1000, Boson Biology of Beijing, China ), PKAα/β (dilution 1:1000, Boson Biology of Beijing, China ), cat Polyclonal (dilution 1:1000, SAB, Zhejiang, China), CREB (dilution 1:1000, SAB, Zhejiang, China), p-CREB (dilution 1:1000, Boson Biology of Beijing, China), IκBα (dilution 1:1000, Beijing Boorson Biological, China), NF-κBP65 (dilution 1:1000, Boster Biological Technology, Wunan, China), β-actin (dilution 1:2000, Boster Biological Technology, Wunan, China), Beta tubulin (dilution 1:2000, Beijing Boorson Biological, China), and GAPDH (dilution 1:5000, Boster Biological Technology, Wunan, China) were used. The antibodies were incubated at 4 ℃ with p-CREB for 12 h, and the other primary antibodies were incubated with 5% skim milk, followed by incubation with goat anti-rabbit anti-mouse secondary antibody (dilution 1:20,000, Boster Biological Technology, Wunan, China). Proteins were visualized using an enhanced chemiluminescence method, and bands of immune responses were visualized using the Omega Lum C Imaging System (Gel Company Inc., San Francisco, CA, USA) and quantified by density measurement using ImageJ software (NIH, Maryland, USA).

### Statistical analysis

GraphPad Prism version 8.0 for Windows (GraphPad Software, La Jolla, California, USA) was used to perform statistical analysis. In all analyses, the confidence level was set at 95%, and *P* < 0.05 was considered statistically significant.

### Supplementary Information

Supplementary Information 1.

Supplementary Information 2.

Supplementary Information 3.

Supplementary Information 4.

Supplementary Information 5.

Supplementary Information 6.

Supplementary Figure S1.

## Data Availability

Supplementary Information is available for this paper. All data generated or analyzed during this study are included in this published article and its supplementary information files.

## References

[1] Liu F (2020). Involvement of NF-κB in the reversal of CYP3A down-regulation induced by sea buckthorn in BCG-induced rats. PLoS ONE.

[2] Zhao S (2020). Protective effect of seabuckthorn berry juice against acrylamide-induced oxidative damage in rats. J. Food Sci..

[3] Valenti L, Pedica F, Colombo M (2022). Distinctive features of hepatocellular carcinoma in non-alcoholic fatty liver disease. Dig. Liver Dis.: Off. J. Italian Soc. Gastroenterol. Italian Assoc. Study Liver.

[4] Wang R (2021). Gut microbiome, liver immunology, and liver diseases. Cell. Mol. Immunol..

[5] Lee HW, Lee JS, Ahn SH (2020). Hepatitis B virus cure: Targets and future therapies. Int. J. Mol. Sci..

[6] Leoni S, Casabianca A, Biagioni B, Serio I (2022). Viral hepatitis: Innovations and expectations. World J. Gastroenterol..

[7] Matsuda M, Seki E (2020). Hepatic stellate cell-macrophage crosstalk in liver fibrosis and carcinogenesis. Semin. Liver Dis..

[8] Dienes HP, Drebber U (2010). Pathology of immune-mediated liver injury. Dig. Dis. (Basel, Switzerland).

[9] Rao T, Liu YT, Zeng XC, Li CP, Ou-Yang DS (2021). The hepatotoxicity of polygonum multiflorum: The emerging role of the immune-mediated liver injury. Acta Pharmacol. Sinica.

[10] Morgan ET, Skubic C, Lee CM, Cokan KB, Rozman D (2020). Regulation of cytochrome P450 enzyme activity and expression by nitric oxide in the context of inflammatory disease. Drug Metab. Rev..

[11] Stanke-Labesque F, Gautier-Veyret E, Chhun S, Guilhaumou R (2020). Inflammation is a major regulator of drug metabolizing enzymes and transporters: Consequences for the personalization of drug treatment. Pharmacol. Ther..

[12] Lenoir C, Rollason V, Desmeules JA, Samer CF (2021). Influence of inflammation on cytochromes P450 activity in adults: A systematic review of the literature. Front. Pharmacol..

[13] Tavares LP (2020). Blame the signaling: Role of cAMP for the resolution of inflammation. Pharmacol. Res..

[14] Hashimoto K, Tsuji Y (2017). Arsenic-induced activation of the homeodomain-interacting protein kinase 2 (HIPK2) to cAMP-response element binding protein (CREB) axis. J. Mol. Biol..

[15] Jin P (2021). INT-777 prevents cognitive impairment by activating Takeda G protein-coupled receptor 5 (TGR5) and attenuating neuroinflammation via cAMP/PKA/CREB signaling axis in a rat model of sepsis. Experim. Neurol..

[16] Negreiros-Lima GL (2020). Cyclic AMP regulates key features of macrophages via PKA: Recruitment, reprogramming and efferocytosis. Cells.

[17] Wang J, Wong YK, Liao F (2018). What has traditional Chinese medicine delivered for modern medicine?. Expert Rev. Mol. Med..

[18] Ciesarová Z (2020). Why is sea buckthorn (*Hippophae rhamnoides* L.) so exceptional? A review. Food Res. Int. (Ottawa, Ont.).

[19] Liu H (2015). Protective effects of sea buckthorn polysaccharide extracts against LPS/d-GalN-induced acute liver failure in mice via suppressing TLR4-NF-κB signaling. J. Ethnopharmacol..

[20] Zielińska A, Nowak I (2017). Abundance of active ingredients in sea-buckthorn oil. Lipids Health Dis..

[21] Li X (2021). West meets east: Open up a dialogue on phytomedicine. Chin. Med..

[22] Ji M, Gong X, Li X, Wang C, Li M (2020). Advanced research on the antioxidant activity and mechanism of polyphenols from hippophae species-a review. Mol. (Basel, Switzerland).

[23] Gao ZL, Gu XH, Cheng FT, Jiang FH (2003). Effect of sea buckthorn on liver fibrosis: A clinical study. World J. Gastroenterol..

[24] He ZX, Chen XW, Zhou ZW, Zhou SF (2015). Impact of physiological, pathological and environmental factors on the expression and activity of human cytochrome P450 2D6 and implications in precision medicine. Drug Metab. Rev..

[25] Pan X, Ning M, Jeong H (2017). Transcriptional regulation of CYP2D6 expression. Drug Metab. Dispos.: Biol. Fate Chem..

[26] Taylor C (2020). A review of the important role of CYP2D6 in pharmacogenomics. Genes.

[27] Grobe N, Kutchan TM, Zenk MH (2012). Rat CYP2D2, not 2D1, is functionally conserved with human CYP2D6 in endogenous morphine formation. FEBS Lett..

[28] Lin Q (2019). NF-κB-mediated regulation of rat CYP2E1 by two independent signaling pathways. PloS one.

[29] Mallick P, Taneja G, Moorthy B, Ghose R (2017). Regulation of drug-metabolizing enzymes in infectious and inflammatory disease: Implications for biologics-small molecule drug interactions. Expert Op. Drug Metab. Toxicol..

[30] Yu A, Haining RL (2001). Comparative contribution to dextromethorphan metabolism by cytochrome P450 isoforms in vitro: Can dextromethorphan be used as a dual probe for both CTP2D6 and CYP3A activities?. Drug Metab. Dispos.: Biol. Fate Chem..

[31] Rüdesheim S, Selzer D, Fuhr U, Schwab M, Lehr T (2022). Physiologically-based pharmacokinetic modeling of dextromethorphan to investigate interindividual variability within CYP2D6 activity score groups. CPT: Pharmacomet. Syst. Pharmacol..

[32] Mitchell JP, Carmody RJ (2018). NF-κB and the transcriptional control of inflammation. Int. Rev. Cell Mol. Biol..

[33] Serezani CH, Ballinger MN, Aronoff DM, Peters-Golden M (2008). Cyclic AMP: Master regulator of innate immune cell function. Am. J. Respir. Cell Mol. Biol..

[34] Aronoff DM, Carstens JK, Chen GH, Toews GB, Peters-Golden M (2006). Short communication: differences between macrophages and dendritic cells in the cyclic AMP-dependent regulation of lipopolysaccharide-induced cytokine and chemokine synthesis. J. Interf. Cytokine Res.: Off. J. Int. Soc. Interf. Cytokine Res..

[35] Takahashi N, Tetsuka T, Uranishi H, Okamoto T (2002). Inhibition of the NF-kappaB transcriptional activity by protein kinase A. Eur. J. Biochem..

[36] Fraser DA, Arora M, Bohlson SS, Lozano E, Tenner AJ (2007). Generation of inhibitory NFkappaB complexes and phosphorylated cAMP response element-binding protein correlates with the anti-inflammatory activity of complement protein C1q in human monocytes. J. Biol. Chem..

[37] Dalli J (2015). The regulation of proresolving lipid mediator profiles in baboon pneumonia by inhaled carbon monoxide. Am. J. Respir. Cell Mol. Biol..

[38] Lee HJ, Ko HJ, Song DK, Jung YJ (2018). Lysophosphatidylcholine promotes phagosome maturation and regulates inflammatory mediator production through the protein kinase A-phosphatidylinositol 3 kinase-p38 mitogen-activated protein kinase signaling pathway during mycobacterium tuberculosis infection in mouse macrophages. Front. Immunol..

[39] Guo WN, Zhu B, Ai L, Yang DL, Wang BJ (2018). Animal models for the study of hepatitis B virus infection. Zool. Res..

[40] Li D (2019). Correction: Glioma-associated human endothelial cell-derived extracellular vesicles specifically promote the tumourigenicity of glioma stem cells via CD9. Oncogene.

[41] Neyshaburinezhad N (2020). Evaluation of hepatic CYP2D1 activity and hepatic clearance in type I and type II diabetic rat models, before and after treatment with insulin and metformin. Daru: J. Fac. Pharm., Tehran Univ. Med. Sci..

