# Supplementary material for: Hippophae rhamnoides reverses decreased CYP2D6 expression in rats with BCG-induced liver injury
Source: Sci Rep. 2023 Oct 13;13:17425. doi: 10.1038/s41598-023-44590-w (PMC10575986; doi:10.1038/s41598-023-44590-w)
Supplement: Supplementary file 1 — Supplementary Information 1. [file 41598_2023_44590_MOESM1_ESM.pdf]

areas with inflammatory cell infiltration (S)	$\bar{x}$	SD
Control	1.34	0.31
HRP	1.34	0.31
BCG	26.94	1.68
BCG+HRP(small)	15.21	1.05
BCG+HRP(medium)	14.14	0.99
BCG+HRP(large)	10.94	0.82

Supplementary file S1: In figure 2 the effect of HRP on liver pathology of rats with BCG-induced immune-mediated liver injury. (G) Representative light microscope images of areas with inflammatory cell infiltration (S) in the liver in each group. Data are expressed as the mean ± standard deviation (SD) (n = 6 rats).
