# Supplementary material for: Hippophae rhamnoides reverses decreased CYP2D6 expression in rats with BCG-induced liver injury
Source: Sci Rep. 2023 Oct 13;13:17425. doi: 10.1038/s41598-023-44590-w (PMC10575986; doi:10.1038/s41598-023-44590-w)
Supplement: Supplementary file 2 — Supplementary Information 2. [file 41598_2023_44590_MOESM2_ESM.pdf]

| liver-to-body-weight | $\bar{x}$ | SD |
|---|---|---|
| Control | 3.60 | 0.39 |
| HRP | 3.78 | 0.36 |
| BCG | 7.60 | 1.55 |
| BCG+HRP(small) | 5.87 | 0.38 |
| BCG+HRP(medium) | 5.67 | 0.58 |
| BCG+HRP(large) | 4.45 | 0.24 |

Supplementary file S2: In figure 3 the effect of HRP on the liver weight of rats with BCG-induced immune-mediated liver injury. Data are expressed as the mean ± SD (n = 6 rats).
