# Supplementary material for: Hippophae rhamnoides reverses decreased CYP2D6 expression in rats with BCG-induced liver injury
Source: Sci Rep. 2023 Oct 13;13:17425. doi: 10.1038/s41598-023-44590-w (PMC10575986; doi:10.1038/s41598-023-44590-w)
Supplement: Supplementary file 3 — Supplementary Information 3. [file 41598_2023_44590_MOESM3_ESM.pdf]

Supplementary file S3
Proteomics profiling

1.1 Protein extraction and digestion

Protein from each sample was mixed with SDT buffer (4%SDS，100mM Tris-HCl，1mM DTT，pH7.6). Protein was digested with trypsin. The digest peptides were desalted with C18 Cartridges (Empore™ SPE Cartridges C18 (standard density), bed I.D. 7 mm, volume 3 ml, Sigma), concentrated by vacuum centrifugation and reconstituted in 40 µl of 0.1% (v/v) formic acid.

1.2 Fractionation (Optional)

Pierce high pH reversed-phase fractionation kit (Thermo scientific) was used to fractionate peptides of each sample by an increasing acetonitrile step-gradient elution according to instructions.

1.3 LC-MS/MS analysis by Q exactive

The peptides were loaded onto a reverse phase trap column (Thermo Scientific Acclaim PepMap100, 100 μm*2 cm, nanoViper C18) connected to the C18-reversed phase analytical column (Thermo Scientific Easy Column, 10 cm long, 75 μm inner diameter, 3μm resin). Buffer A is 0.1% formic acid acetonitrile aqueous solution (2% acetonitrile) and buffer B is 0.1% formic acid acetonitrile aqueous solution (84% acetonitrile) at a flow rate of 300 nl/min controlled by IntelliFlow technology. Samples were analyzed by Q-Exactive MS. Data was acquired using a data-dependent top10 method dynamically choosing the most abundant precursor ions from the survey scan (300–1800 m/z) for higher energy collision dissociation (HCD) fragmentation. Survey scans were acquired at a resolution of 70,000 at m/z 200 and resolution for HCD spectra was set to 17,500 at m/z 200. Normalized collision energy was 30 eV and the underfill ratio was defined as 0.1%. The instrument was run with peptide recognition mode enabled.

1.4 Identification and quantitation of proteins

MaxQuant software (version 1.5.3.17) was used in the MS data analysis for identification and quantitation analysis. MS data were searched against the Uniport. The search was performed by the enzymatic cleavage rule of Trypsin. Methionine oxidation and protein N-terminal acetylation modification was included as variable modification, and carbamidomethyl was set as fixed modification. The value of false discovery rate for peptide and protein identification was set to 0.01.

1.5 Bioinformatic analysis

The motifs were analyzed by MeMe (http://meme-suite.org/index.htm). We extracted the amino acid sequences that contained the modified site and six upstream/downstream amino acids from the modified site (totally 13 amino acid sites). These sequences were used to predicted motifs in this study (parameters: width: 13, occurrences: 20, background: rats).
