# Supplementary material for: Hippophae rhamnoides reverses decreased CYP2D6 expression in rats with BCG-induced liver injury
Source: Sci Rep. 2023 Oct 13;13:17425. doi: 10.1038/s41598-023-44590-w (PMC10575986; doi:10.1038/s41598-023-44590-w)
Supplement: Supplementary file 4 — Supplementary Information 4. [file 41598_2023_44590_MOESM4_ESM.pdf]

fig5A

T(h)	Control $\bar{x}$	Control SD	BCG $\bar{x}$	BCG SD	BCG+HRP(100mg•kg-1) $\bar{x}$	BCG+HRP(100mg•kg-1) SD
0	0.00	0.00	0.00	0.00	0.00	0.00
0.25	91.51	25.29	111.17	24.84	123.32	24.85
0.5	83.19	22.99	179.78	45.09	99.70	27.13
1	69.33	19.16	147.17	32.88	83.47	15.01
2	49.52	13.69	105.12	23.49	61.02	11.10
4	22.58	2.13	58.40	13.05	30.82	5.60
6	6.65	8.82	32.44	7.25	17.12	3.11
8	0.67	1.03	19.10	6.11	0.83	0.98
12	0.17	0.41	6.51	2.92	0.50	0.84

fig5B

AUC	$\bar{x}$	SD
Control	241.13	71.08
BCG	615.25	140.02
BCG+HRP(100mg•kg-1)	321.72	56.68

CL	$\bar{x}$	SD
Control	0.067	0.017
BCG	0.025	0.006
BCG+HRP(100mg•kg-1)	0.048	0.008

Cmax	$\bar{x}$	SD
Control	91.51	25.29
BCG	179.78	45.09
BCG+HRP(100mg•kg-1)	123.32	24.85

MRT	$\bar{x}$	SD
Control	2.05	0.24
BCG	3.15	0.09

BCG+HRP(100mg•kg-1)	2.35	0.07

Vd	$\bar{x}$	SD
Control	0.03	0.01
BCG	0.08	0.02
BCG+HRP(100mg•kg-1)	0.06	0.05

T1/2	$\bar{x}$	SD
Control	0.37	0.11
BCG	2.28	0.20
BCG+HRP(100mg•kg-1)	0.90	0.73

**fig5C**	$\bar{x}$	SD
Control	107.83	9.87
HRP	107.17	7.88
BCG	60.17	8.13
BCG+HRP(small)	83.90	8.64
BCG+HRP(medium)	90.60	9.43
BCG+HRP(large)	105.07	7.53

Supplementary file S4: In figure 5 the effect of HRP on the metabolic activity of CYP2D6 in rats with BCG-induced immune-mediated liver injury. (A) Blood samples were collected 0.25, 0.5, 1, 2, 4, 6, 8, and 12 h after DM administration. (B) Parameters (AUC, CL, Cmax, MRT, Vd and T1/2) were calculated from the plasma dextromethorphan concentration curve as described. T1/2, half-life; Cmax, peak concentration; CL, clearance rate; AUC, area under the curve; MRT, internal dwell time; Vd, apparent volume of distribution. (C) Metabolic rate was expressed by the amount of dextromethorphan metabolized after incubation with microsomes in vitro and was normalized by the control group. Data is expressed as the mean ± SD (n = 6 rats).
