# Supplementary material for: Hippophae rhamnoides reverses decreased CYP2D6 expression in rats with BCG-induced liver injury
Source: Sci Rep. 2023 Oct 13;13:17425. doi: 10.1038/s41598-023-44590-w (PMC10575986; doi:10.1038/s41598-023-44590-w)
Supplement: Supplementary file 5 — Supplementary Information 5. [file 41598_2023_44590_MOESM5_ESM.pdf]

2D6	$\bar{x}$	SD
Control	1.00	0.00
HRP	1.02	0.05
BCG	0.55	0.07
BCG+HRP(small)	0.56	0.05
BCG+HRP(medium)	0.85	0.14
BCG+HRP(large)	0.88	0.12

PKA	$\bar{x}$	SD
Control	1.00	0.00
HRP	0.94	0.11
BCG	0.53	0.06
BCG+HRP(small)	0.69	0.03
BCG+HRP(medium)	0.80	0.09
BCG+HRP(large)	0.93	0.14

NF-κB	$\bar{x}$	SD
Control	1.00	0.00
HRP	1.05	0.08
BCG	1.97	0.38
BCG+HRP(small)	1.96	0.44
BCG+HRP(medium)	1.84	0.36
BCG+HRP(large)	1.01	0.23

IκB	$\bar{x}$	SD
Control	0.99	0.02
HRP	1.10	0.12
BCG	0.55	0.04
BCG+HRP(small)	0.69	0.13
BCG+HRP(medium)	0.94	0.15
BCG+HRP(large)	1.09	0.26

P-CREB-to-CREB protein intensity ratio	$\bar{x}$	SD
Control	100.00	0.00
HRP	90.87	14.99
BCG	26.96	4.75
BCG+HRP(small)	41.14	2.51
BCG+HRP(medium)	64.91	14.64
BCG+HRP(large)	101.89	15.72

Supplementary file S5: In figure 6 the effect of HRP on the expression of CYP2D6, PKA, CREB, PCREB, IκB, and NF-κB in rats with BCG-induced immune-mediated liver injury. Liver proteins were extracted to determine the expression levels of CYP2D6, PKA, CREB, PCREB, IκB, and NF-κB. SDS-PAGE was performed using equal amounts (30 μg) of protein, and western blotting was performed using antibodies against CYP2D6, PKA, CREB, PCREB, i-κB, and NF-κB. The results were normalized to tubulin, GAPDH or β-actin. The protein expression levels of CYP2D6 (A), PKA (B), IκB and NF-κB (C), CREB, and PCREB (D) in the rat liver were measured by western blotting. The expression levels of CYP2D6, PKA, IκB, NF-κB, CREB, and PCREB were quantified using the ImageQuant analysis software (GE Healthcare Life Sciences, Little Chalfont, UK). The data represent the mean ± SD of three independent experiments.
