# Supplementary material for: Hippophae rhamnoides reverses decreased CYP2D6 expression in rats with BCG-induced liver injury
Source: Sci Rep. 2023 Oct 13;13:17425. doi: 10.1038/s41598-023-44590-w (PMC10575986; doi:10.1038/s41598-023-44590-w)
Supplement: Supplementary file 6 — Supplementary Information 6. [file 41598_2023_44590_MOESM6_ESM.pdf]

CAMP	$\bar{x}$	SD
Control	7. 90	0. 41
HRP	7. 93	0. 50
BCG	5. 75	0. 82
BCG+HRP(small)	5. 78	1. 33
BCG+HRP(medium	6. 75	1. 44
BCG+HRP(large)	8. 02	0. 94

TNF-α	$\bar{x}$	SD
Control	283. 65	12. 34
HRP	288. 77	10. 30
BCG	331. 00	5. 10
BCG+HRP(small)	318. 69	2. 97
BCG+HRP(medium	288. 32	20. 95
BCG+HRP(large)	277. 67	25. 06

1L-1β	$\bar{x}$	SD
Control	27. 98	2. 26
HRP	28. 56	2. 42
BCG	44. 87	3. 61
BCG+HRP(small)	42. 08	3. 38
BCG+HRP(medium	36. 48	2. 93
BCG+HRP(large)	30. 88	2. 48

Supplementary file S6: In figure 7 the effect of HRP on cAMP, TNF-α, and IL-1β levels in rats with immune-mediated liver injury. Rats were administered BCG (125 mg kg-1 intravenously, once every two weeks) or BCG +HRP (50, 100, or 200 mg kg-1 d-1 orally for 13 d). Each bar represents mean ± SD of three independent experiments (n = 10 per group).
