# Supplementary material for: Hippophae rhamnoides reverses decreased CYP2D6 expression in rats with BCG-induced liver injury
Source: Sci Rep. 2023 Oct 13;13:17425. doi: 10.1038/s41598-023-44590-w (PMC10575986; doi:10.1038/s41598-023-44590-w)
Supplement: Supplementary file 7 — Supplementary Figure S1. [file 41598_2023_44590_MOESM7_ESM.pdf]

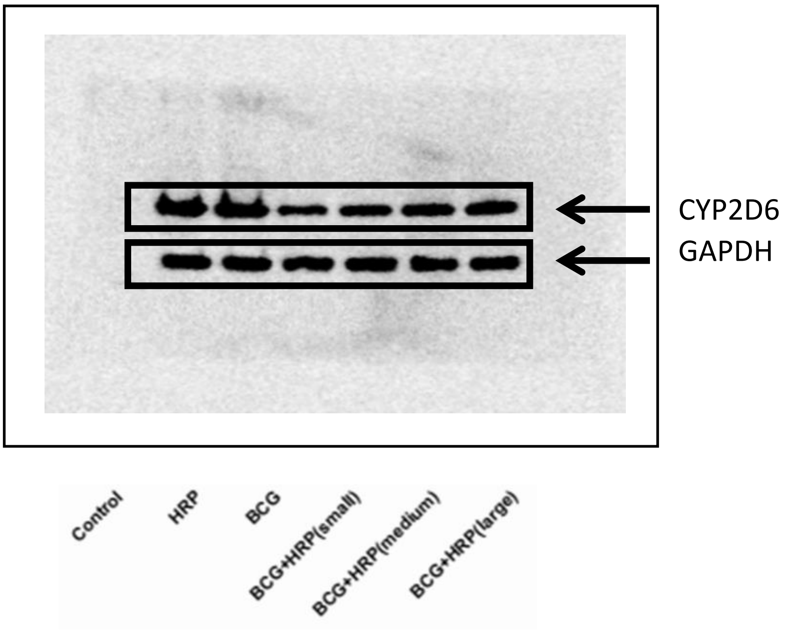

Supplementary figure S1

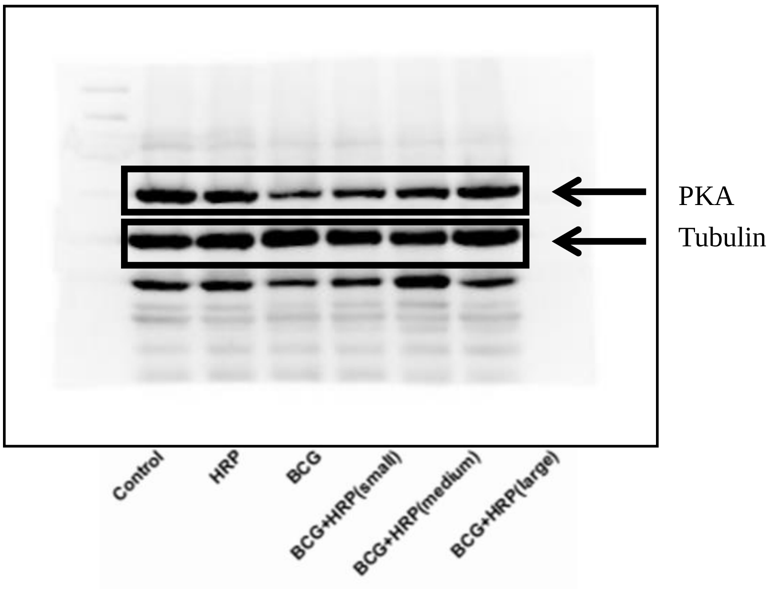

Supplementary figure S2

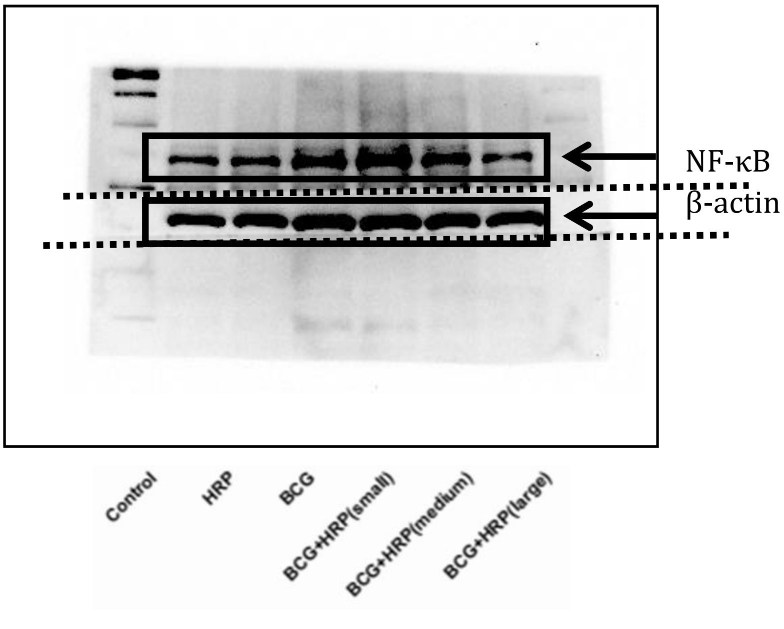

Supplementary figure S3-1

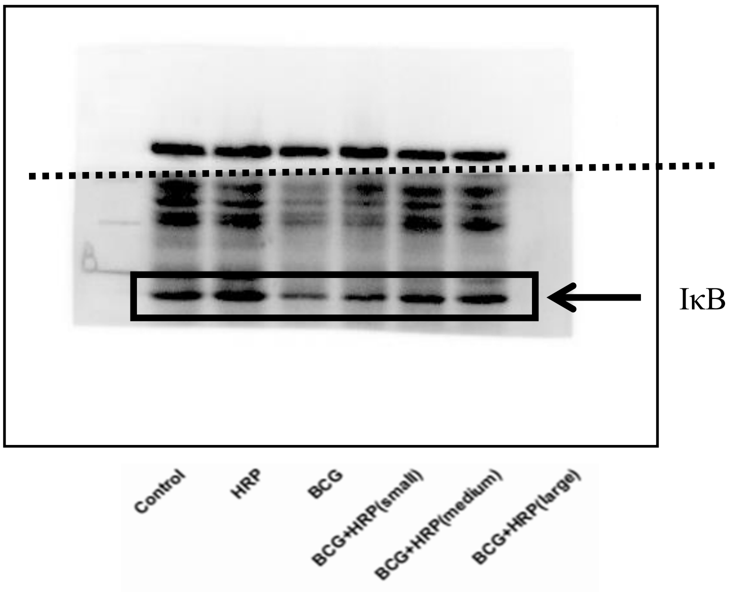

Supplementary figure S3-2

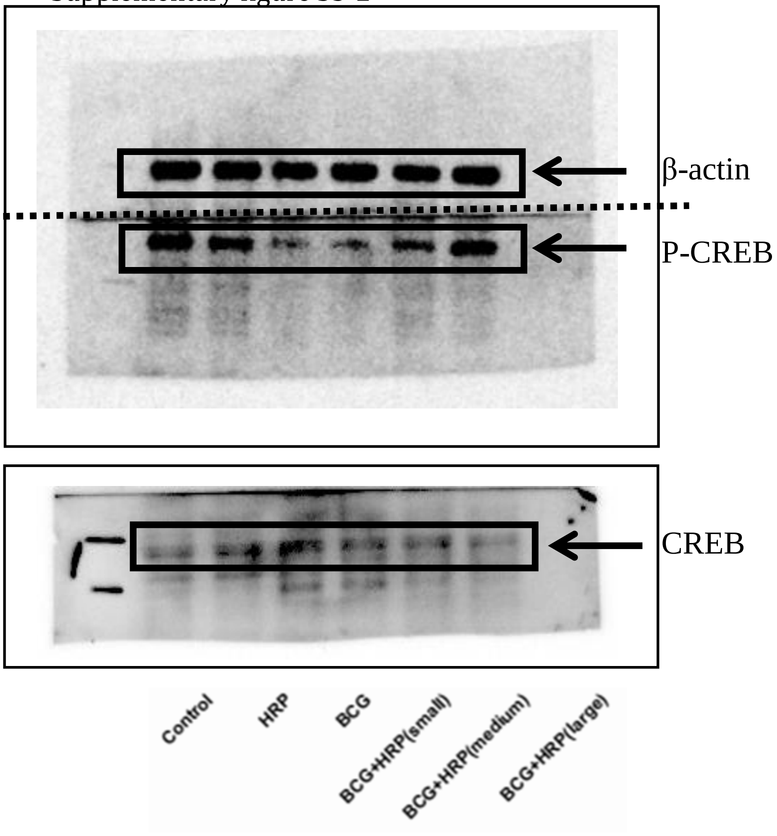

Supplementary figure S4

Supplementary figure S1-4: In figure 6 the effect of HRP on the expression of CYP2D6, PKA, CREB, PCREB, IκB, and NF-κB in rats with BCG-induced immune-mediated liver injury. Rats were administered BCG (125 mg kg^{-1} intravenously, once for two weeks) or BCG + HRP (50, 100, or 200 mg kg^{-1} d^{-1} orally for seven days). Liver proteins were extracted to determine the expression levels of CYP2D6, PKA, CREB, PCREB, IκB, and NF-κB. SDS-PAGE was performed using equal amounts (30 μg) of protein, and western blotting was performed using antibodies against CYP2D6, PKA, CREB, PCREB, i-κB, and NF-κB. The results were normalized to tubulin, GAPDH or β-actin. The protein expression levels of CYP2D6 (A), PKA (B), IκB and NF-κB (C), CREB, and PCREB (D) in the rat liver were measured by western blotting. The expression levels of CYP2D6, PKA, IκB, NF-κB, CREB, and PCREB were quantified using the ImageQuant analysis software (GE Healthcare Life Sciences, Little Chalfont, UK). The data represent the mean ± SD of three independent experiments.
